# Socioeconomic disparities in vestibular schwannoma diagnosis and management: A systematic review

**DOI:** 10.1007/s10143-026-04478-5

**Published:** 2026-09-22

**Authors:** Anne R. Lally, Rachel L. Welch, Anthony Terraciano, Taylor Vadset, Sabrina Saidpour, Rachel Rayden, Elizabeth Cohen, Ashley Choi, Shaila Berlas, Manish Bhatta, Sayak R. Ghosh, Vijay Agarwal

**Affiliations:** 1 Department of Neurological Surgery, University of Washington, Seattle, WA USA; 2 Albert Einstein College of Medicine Bronx, Bronx, NY USA; 3 Department of Neurological Surgery, Montefiore/Einstein, Bronx, NY USA

**Keywords:** Vestibular schwannoma, Social determinants of health, Outcomes, Disparities, Quality of life

## Abstract

Disparities in neurosurgical care are increasingly recognized, yet how they affect the diagnosis and management of vestibular schwannoma (VS) remains unclear. Differences in tumor size at diagnosis, treatment selection, and outcomes may be influenced by race, insurance status, income, and geography. To date, no comprehensive synthesis has addressed these inequities in patients with sporadic VS. To systematically evaluate the literature for evidence of social determinants of health (SDoH) in the diagnosis, treatment, and outcomes of patients with sporadic vestibular schwannoma. We conducted a PRISMA-guided systematic review of PubMed, Embase, and Web of Science (2000–2024). Included studies examined adult patients with sporadic VS and reported at least one socioeconomic variable. Non-English studies and those involving pediatric patients were excluded. Studies focusing on neurofibromatosis type 2 were excluded. Screening and data extraction were conducted independently by multiple reviewers using Covidence. Outcomes of interest included tumor size, treatment modality (surgery, radiosurgery, observation), hearing preservation, complications, and recurrence. Study quality was assessed using the GRADE framework and evaluated across the domains of risk of bias, inconsistency, indirectness, imprecision, and publication bias. Of 487 records identified, 40 met inclusion criteria. Most were US-based, retrospective studies that utilized national databases for patient recruitment. Race and insurance status were the most frequently reported variables; income and geographic indicators were less commonly assessed. Outcomes described the likelihood of treatment received, including surgery or SRS, complications including morbidity and mortality, length of stay, and discharge disposition including rehab and readmission. Few studies assessed functional status (*n*=2), hearing outcomes (*n*=2), or quality of life (n=1). In total, 358,843 patients were included across the 40 studies analyzed. This review suggests that disparities in vestibular schwannoma care are multi-factorial, with race and insurance status showing the most consistent associations between management, outcomes, and disparities. As treatment continues to evolve and become more centralized, future work should prioritize multi-institutional studies to incorporate patient-level socioeconomic data and evaluate outcomes beyond management selection.

## Introduction

Vestibular schwannoma (VS), a benign tumor of the vestibulocochlear nerve, is the third most common benign intracranial tumor and represents 85% of cerebellopontine angle tumors [1]. Typically presenting with unilateral hearing loss and tinnitus [2], most cases are sporadic; bilateral involvement is pathognomonic for neurofibromatosis type 2 (NF2) [1, 3]. Diagnosis is confirmed by magnetic resonance imaging (MRI). Although histologically benign, VS can cause significant morbidity due to mass effect on the brainstem and cerebellum, leading to cranial nerve deficits, vertigo, headache, imbalance, facial paresthesia, and hydrocephalus [4]. In advanced cases, VS can be life-threatening from brainstem compression. While MRI has enabled earlier detection [5], unequal access to advanced imaging may delay diagnosis and stratify outcomes based on socioeconomic or geographic factors. VS also causes underrecognized symptoms of fatigue, anxiety, and imbalance, which may impact patient quality of life (QoL) [6]. Studies show that VS patients report lower QoL scores than non-VS patients [6], supporting the need for routine QoL assessments and supportive interventions.

VS has a greater prevalence than historically recognized, with U.S. incidence estimated at 1.2 per 100,000 annually and lifetime risk exceeding 1 in 500 [2]. Over the past four decades, incidence has risen significantly from 3 to 34 cases per million annually, likely due to improved diagnostic imaging and increased disease awareness, screening protocols, and detection in older adults [3, 5]. Incidence is believed to peak between 65 and 74 years [7] and is comparable between sexes. Yet VS is more commonly diagnosed in White patients than Black, Hispanic, and Asian patients [8, 9], suggesting possible disparities in access to imaging or specialist care.

Standard treatment options for VS include observation, stereotactic radiosurgery (SRS), and surgical resection, with selection based on tumor size, growth rate, and patient context. Observation is often appropriate for small, asymptomatic, or slow-growing tumors, which are stable within the first five years after diagnosis [10]. SRS is used for medium-sized tumors without mass effect and provides better facial and cochlear nerve preservation, while surgical resection is indicated for larger, compressive tumors causing progressive symptoms [11]. Insurance status, income, race, and place of residence have been associated with differences in treatment modality, with underserved groups less likely to receive SRS or surgery [9, 12, 13].

The influence of disparities on the diagnosis and management of VS remains poorly characterized. Emerging evidence suggests that social determinants of health (SDoH), including race, insurance status, income, and geographic location, may affect tumor size at diagnosis, treatment selection, and outcomes [12]. Additionally, patients with lower income or without insurance report significantly worse QoL at the time of diagnosis [12], potentially reflecting limited access to specialty care. However, most studies are retrospective and not able to comprehensively evaluate the cumulative effects of SDoH on long-term clinical outcomes. Additionally, no studies to date have thoroughly examined the influence of SDoH on the decision-making process across different stages of care, including surveillance, intervention timing, and access to emerging therapies. This systematic review presents the first comprehensive synthesis of disparities in VS care. By highlighting strengths and gaps in the literature on these inequities, it aims to guide future equity-focused research and inform more inclusive clinical practices in skull base neurosurgery.

## Methods

### Guidelines/sources

Following PRISMA guidelines, we searched PubMed, Embase, and Web of Science (2000–2024). To identify relevant articles, a search algorithm containing the following terms was used: (“vestibular schwannoma” OR “acoustic neuroma”) AND (disparities OR inequity OR inequality OR race OR ethnicity OR socioeconomic OR insurance OR “access to care” OR geographic OR rural OR urban OR regional). 487 studies were imported for screening, and 165 were removed as duplicates.

### Study selection and inclusion/exclusion criteria

The authors included studies examining retrospective and prospective adult patient cohorts with sporadic vestibular schwannomas/acoustic neuromas and reporting at least one socioeconomic variable including race/ethnicity, age, sex, insurance status, income level, and geographic location. Exclusion criteria included pediatric patient populations, unless explicitly reported as a subgroup, studies focused on patients with neurofibromatosis type 2, studies that did not stratify outcomes based on socioeconomic factors, non-English language publications, and grey literature. Because several retrospective and database-based studies did not explicitly report exclusion of neurofibromatosis patients, this information was recorded during study selection and considered during interpretation of the findings.

Two independent, blinded reviewers screened abstracts and full texts, with a third resolving discrepancies. When full text was unavailable, the authors utilized interlibrary loan. 322 abstracts were screened, and 227 studies were excluded. Of the 95 full-text articles assessed, 40 were deemed eligible for data collection.

### Data collection

Two independent reviewers extracted and cross-checked data to reach consensus about each study. Outcomes assessed included tumor size at diagnosis, treatment modality, hearing preservation rates, post-treatment complications and neurological deficits, and overall survival or recurrence rates.

### Evidence appraisal

Because the included studies were predominantly retrospective analyses and were heterogeneous in design, patient population, and outcomes assessed, we used the GRADE (Grading of Recommendations, Assessment, Development and Evaluations) framework [14] to assess limitations in the evidence base. Each study was evaluated across the domains of risk of bias, inconsistency, indirectness, imprecision, and publication bias. Studies were then categorized as very low, low, moderate, or high certainty, and these ratings were interpreted cautiously given the nature of the literature. Specifically, under the GRADE framework, observational and retrospective studies begin at a low certainty level. Thus, this framework was selected to provide a standardized and conservative assessment across the five evaluation domains.

## Results

Of 40 included studies, 95% (*n* = 38) were retrospective and 70% (*n* = 28) utilized national databases (Table 1); 65% (*n* = 26) did not explicitly report NF2 exclusion. Assessed variables included race/age (*n* = 27 each), sex (*n* = 24), insurance (*n* = 23), income (*n* = 20), and geography (*n* = 16). Primary outcomes were treatment modality (*n* = 19), complications (*n* = 13), discharge disposition (*n* = 16), and length of stay (*n* = 9). Few studies assessed functional status (*n* = 2), hearing outcomes (*n* = 2), or quality of life (*n* = 1). In total, 358,843 patients were included across the 40 studies analyzed.

**Table 1:**
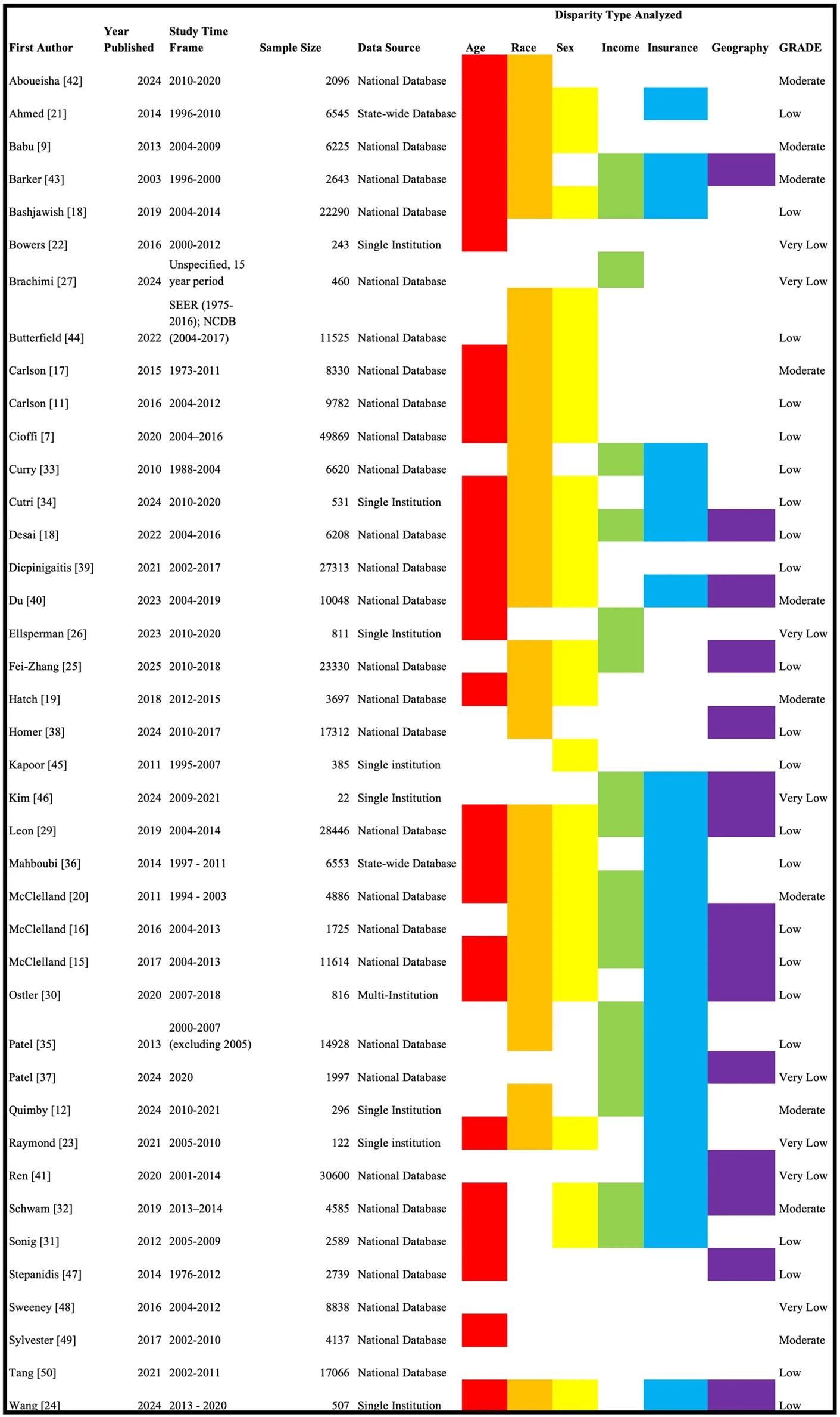
Disparity type of included studies

### Race/ethnicity

Of the 27 studies assessing race, most utilized national databases, and the most commonly assessed outcome was treatment (Tables 2 and 3). Based on the GRADE criteria, 1 study was rated as very low, 17 as low, and 8 as moderate certainty of evidence.

**Table 2:** Study Characteristics

	**N (%)**
Total Number of Studies	40
Type of Study	
Retrospective	38 (95.0)
Retrospective case-control	1 (2.5)
Prospective cohort	1 (2.5)
Data Sources	
National Database	28 (70.0)
International Database	1 (2.5)
State Database	2 (5.0)
Multi-institution	1 (2.5)
Single Institution	8 (20)
Disparity Type	
Race/Ethnicity	27
Sex	24
Income	19
Insurance	23
Geography	18
Age	27

**Table 3:** Outcomes Examined by Disparity

Race/Ethnicity (n=27)	
Treatment	14
Complications	10
Discharge Disposition	5
Length of Stay	5
Tumor Characteristics	2
Followup	2
Hearing	1
QoL	1
Incidence	1
Sex (n=24)	
Treatment	13
Complications	3
Discharge Disposition	4
Length of Stay	3
Tumor Characteristics	2
Followup/Readmission	3
Mortality	4
Age (n=27)	
Treatment	11
Complications	5
Discharge Disposition	8
Length of Stay	4
Tumor Characteristics	2
Follow up	1
Geography (n=16)	
Treatment	11
Complications	2
Discharge Disposition	5
Length of Stay	3
Tumor Characteristics	1
Followup	2
Incidence	1
Income (n=20)	
Treatment	12
Complications	5
Discharge Disposition	4
Length of Stay	2
Tumor Characteristics	2
Followup	3
QoL	1
Insurance (n=23)	
Treatment	7
Complications	5
Discharge Disposition	6
Length of Stay	5
QoL	1

Several studies reported that Black and/or Hispanic patients were less likely to be recommended surgery and more likely to be managed with observation. For example, Babu et al. reported that Black patients were half as likely to undergo surgical resection and nearly twice as likely to undergo observation as compared to White patients, despite presenting with larger tumors. McClelland et al. [15] and McClelland et al. [16] similarly reported that Black race was independently associated with observation over surgical intervention. Although Carlson et al. [11, 17] also suggested that Black patients were less likely to receive surgery, the authors found that Hispanic patients were more likely to undergo resection compared to non-Hispanic White patients. In contrast, Desai et al. reported no independent effect of race after adjusting for geography and income.

When studies adjusted for socioeconomic factors, some associations by race were attenuated. Quimby et al. demonstrated that only insurance and income were significant in multivariable models identifying postoperative QoL. Fei-Zhang et al. similarly found that low socioeconomic status of the patient’s neighborhood was a stronger predictor of mortality and treatment delays than race or rurality.

### Sex

Of the 24 studies assessing sex disparities in VS care, most utilized national databases and assessed management (Tables 2 and 3). One study was rated very low, 17 were low, and 6 were rated as moderate level of certainty.

Cioffi et al. found no difference in VS incidence by sex, and most studies found no sex-based differences in initial VS management [9, 11, 18]. While most studies did not identify differences in management related to sex, some studies described differences in post-operative outcomes including length of stay, non-routine discharge, and all-cause mortality. Additionally, Du et al. reported no association between sex and access to high-volume centers. However, McClelland et al. 2016 reported that male sex was independently associated with receipt of radiation among geriatric patients (OR 1.3, *p* = 0.03). Additionally, Kapoor et al. observed that 90.9% of SRS treatment failures occurred in female patients (*p* = 0.03).

In studies of post-operative outcomes, Hatch et al. found higher complication and readmission rates in male patients. While Dicpinigaitis et al. found that female sex was associated with lower odds of non-routine discharge (OR 0.74, 95% CI 0.63–0.87, *p* < 0.001), Sonig et al. reported that female patients were more likely to be discharged to a facility compared to males (OR 1.34, 95% CI 1.06–1.70, *p* = 0.016). Although Schwam et al. found that female sex was associated with prolonged hospital stay (*p* = 0.001), Fei-Zhang et al. reported that female sex was independently associated with lower all-cause mortality (HR 0.77, 95% CI 0.70–0.84, *p* < 0.001).

### Age

Twenty-seven studies assessed age disparities in VS care. The majority of these utilized national databases and examined the association of age with management modality, including observation and surgery, and discharge disposition or readmission most frequently. Using the GRADE criteria to assess certainty of evidence, 3 studies were rated very low, 15 were rated low, and 9 were rated moderate certainty.

Nine studies found that older patients were more likely to be managed with observation than surgery. Although older age is historically associated with conservative management, this trend may not be clinically significant [9]. Among patients undergoing SRS for VS treatment, gamma knife was used more often when compared to linear accelerator in younger patients [18]. Increasing age was also associated with longer post-operative ICU length of stay [19].

While Dicpinigaitis et al. found that increasing age was significantly associated with routine discharge, other studies reported that increasing age was significantly associated with adverse discharge disposition [20, 21]. Schwam et al. and Hatch et al. found that age was not consistently associated with increased readmission, yet age was associated with discharge to a facility and prolonged length of stay. Furthermore, Bowers et al. reported that there was no difference in incidence of complications between older and younger age groups, while Hatch et al. observed significantly higher complication and mortality rates in older adults. Lastly, Bowers et al. [22] found that geriatric patients were more likely than younger patients to report facial numbness and dizziness. Radiographically, Ostler et al. (2020) reported that older age was associated with smaller tumor size.

### Geography

Sixteen studies analyzed the relationship between geographic variables and VS care. Most studies utilized national databases and commonly assessed outcomes such as treatment, discharge disposition, and length of stay. Geographic factors included urban versus rural residence (*n* = 9), region of home or hospital (*n* = 6), and distance from treatment center (*n* = 4). Overall, certainty of evidence was low, with 4 studies rated as very low certainty, 10 as low certainty, and 2 as moderate certainty.

Desai et al. found that rural zip codes and zip codes in the southern United States (US) were associated with treatment management by LINAC, rather than gamma knife. Leon et al. and Ostler et al. also reported that patients living farther from the treating center were more likely to receive treatment over surveillance. In contrast, Du et al., Kim et al., and Patel et al. found no significant differences in treatment or management based on rurality or distance.

McClelland et al. [16] reported that patients in the central US were more likely to undergo surgery and less likely to receive radiation. Sonig et al. found that the living in the northeastern, southern, and midwestern US was associated with higher likelihood of routine discharge compared to living in the western US.

Additional outcomes assessed related to geography were discharge disposition, complications, follow-up adherence, and diagnosis rates. Schwam et al. found that rural patients had lower odds of readmission yet had a prolonged length of stay compared to urban patients. However, Patel et al. reported that when a psychiatric comorbidity was present, no difference was observed in readmission when comparing rural and urban residence. Sonig et al. found that hospitals located in the Western U.S. were less likely to have non-routine discharges. Single-institution studies [23, 24] found no association between distance from treatment center and adherence to long-term surveillance.

### Income and area-level socioeconomic disparities

Twenty studies examined the relationship between income or area-level socioeconomic status and the management of VS; however, the included studies utilized distinct measurement instruments. All studies utilized area-based socioeconomic proxies referencing zip-code-level median household income derived from US census data, with one study analyzing individual-level income directly [12]. Four studies used area-level deprivation indices, including the Yost Index, Area Deprivation Index (ADI), and Scottish deprivation scores [24–27]. Most studies utilized national databases, and the most assessed outcome was treatment. Overall, certainty of evidence was low, with 6 studies rated as very low, 11 as low, and 3 as moderate certainty.

Although multiple studies found no significant relationship between income and management, findings were not consistent across studies [15, 16, 28–30]. Desai et al. found that patients from low-income areas were more likely to be treated with LINAC over gamma knife. Du et al. reported that patients with incomes below the national median were less likely to receive care at a high-volume center, and similarly, Barker et al. found that patients from higher-income ZIP codes were more likely to receive care from high-volume centers. Additionally, Fei-Zhang et al. found that lower neighborhood socioeconomic status was associated with higher rates of surgery and lower rates of radiation. Similarly, Ellsperman et al. found that higher ADI, or lower socioeconomic status, was associated with more frequent surgical recommendations.

No consistent relationship was observed between income and adherence to follow-up care [23, 24]. However, higher rates of non-routine discharge and prolonged length of stay were associated with lower-income patients [31, 32]. While most studies analyzing morbidity did not identify an association between neighborhood income and postoperative morbidity [20, 33], Ellsperman et al. found that higher ADI, or lower socioeconomic status, was associated with larger and more complex tumors. Further, Fei-Zhang et al. found that lower socioeconomic status was associated with higher all-cause mortality. Using census-based estimates of income, Quimby et al. similarly found that patients in higher income quintiles on univariate and multivariate analysis had significantly better QoL scores at diagnosis.

### Insurance

Twenty-three studies assessed the relationship between insurance status and VS. Most studies used large databases, with treatment and discharge disposition assessed most frequently. Using this appraisal approach, 5 studies were rated as very low certainty, 13 as low certainty, and 5 as moderate certainty.

Multiple studies found that patients with private insurance were more likely to receive surgery or radiation than surveillance [15, 16, 28, 29]. In addition, Medicaid and uninsured patients were more likely to undergo observation or receive care at safety-net hospitals [34]. Among those receiving SRS, patients with private insurance were more likely to receive gamma knife rather than LINAC [18]. However, Ostler et al. found no significant association between insurance status and treatment choice.

In some studies, insurance was also associated with differences in discharge and length of stay. Patients with Medicaid or Medicare were less likely to have routine discharge and more likely to experience prolonged hospitalization [21, 31, 32, 35]. Non-private insurance was also associated with higher complication rates [20, 35, 36], although Barker et al. and McClelland et al. 2017 did not identify a significant association. Higher readmission rates were observed in patients with public or no insurance than among those with private insurance [32, 37].

Quimby et al. found that uninsured patients had lower QoL scores compared to those with commercial insurance. Wang et al. reported reduced follow-up adherence among privately insured patients, while Raymond et al. found no significant association. Medicaid expansion was associated with a higher incidence of VS diagnoses, particularly in low-SES counties and among Black patients [38]. Ren et al. observed no change in surgical admission trends by insurance status.

## Discussion

Vestibular schwannoma management takes place within tertiary academic medical centers. Although centralization of care may improve care coordination and access to specialized imaging and surgical management, it can create barriers for rural and low-income patient populations in transportation, insurance coverage, and access. Thus, we hypothesized that VS care would be impacted by race, sex, age, geography, income, and insurance. This is the first study that systematically reviews the influence of these SDoH domains on VS care.

Of the 40 articles analyzed, which were analyzed as having low certainty of evidence due to their use of large databases, over half assessed race, sex, age, and insurance status, while less than half assessed geography and income. While large administrative databases allow for identification of broad trends across patients, single-institution studies allow for more in-depth examination of individual patient records and tumor characteristics. However, they have less power than larger database studies. Additionally, because 80% of these studies ended before 2020, their generalizability to current practice may be limited. These older studies may not fully represent either the current state of VS care or of demographic disparities.

Studies assessing race and/or ethnicity most consistently demonstrated associations with disparities of care. Database studies frequently reported that Black patients were less likely to undergo surgery and were more often managed with observation even when presenting with larger tumors [9, 11, 15–17]. However, Desai et al. [18] observed that these associations were diminished after adjusting for geography and income, which suggests socioeconomic status and other factors rather than race alone may also affect disparities of care. Quimby et al. [12] and Fei-Zhang et al. [25] suggested that neighborhood socioeconomic status and insurance were stronger predictors of outcomes than race itself. Racial disparities may thus be best understood when contextualized alongside socioeconomic and systemic factors, as it remains difficult to evaluate these as distinct variables given the overlapping influence on access and outcomes.

Most studies found no sex-based disparities in initial management [9, 11, 18], though associations were observed regarding postoperative length of stay, discharge, and mortality [25, 32]. These findings suggest that subtle sex-based differences may also exist in recovery and long-term outcomes, though reliance on large-scale retrospective data limits the ability to assess definitive causality.

While older age frequently correlated with conservative management [9, 20], baseline frailty may serve as a more robust outcome predictor than chronological age alone [39]. Given conflicting reports in this literature on discharge disposition and complications, future studies should focus on further characterizing the intertwined influences of age, comorbidities, and frailty on clinical outcomes.

Geographic factors demonstrated inconsistent associations with management selection across the reviewed studies. Some studies suggested that rural residence or greater distance from a treatment center correlated with higher odds of undergoing surgery or SRS rather than surveillance [29, 30]. Desai et al. [18] observed that patients from rural regions were more likely to undergo LINAC than gamma knife as treatment. However, these trends were not universal, as other studies did not identify significant associations between rurality or distance and tumor management selection [37, 40]. Such variations likely reflect the complex interplay of institutional practice styles and the ongoing centralization of VS care within tertiary academic medical centers. While centralization aims to optimize care coordination, it can introduce structural barriers, like transportation and limited access to specialized imaging, that could influence management patterns in ways that remain difficult to decouple from clinical factors using the current evidence [41].

Multiple retrospective analyses using zip-code-level proxies for income reported no significant association between area-level income and initial treatment selection [16, 30]. However, other studies, such as Fei-Zhang et al. [25], observed an association between lower neighborhood-level socioeconomic status with delays in treatment. These heterogeneous findings likely reflect the inherent limitations of using area-based socioeconomic proxies, which may not precisely represent an individual’s financial status and often obscure the intwined influences of insurance status and geographic access. Consequently, using more granular patient-level data is essential to better characterize the impact of economic status on access to high-volume centers, which may be a better proxy of disparity rather than income alone.

The literature reviewed frequently demonstrated associations between insurance status and variations in VS management, outcomes, and follow-up. These retrospective analyses often observed that patients with private insurance were more likely to undergo surgery or radiosurgery, whereas those with Medicaid or no insurance were more often managed with observation or received care at safety-net hospitals [16, 34]. Insurance type further predicted hospital course and outcomes. Insurance type further served as a predictor in several datasets. For example, publicly insured patients were reported to experience higher rates of prolonged hospitalization, non-routine discharge, and perioperative complications [31, 32, 35]. Additionally, Medicaid expansion was linked to increased VS diagnoses in underserved patients [38]. These findings likely reflect the complex interplay between insurance coverage and access to high-volume specialty centers, which remains challenging to fully decouple from other socioeconomic factors using the current database-level evidence in the literature.

The current literature is limited by reliance on large databases, area-level proxies for socioeconomic status, and lack of data on patient-centered outcomes such as QoL and hearing preservation. The GRADE-informed evidence appraisal should also be interpreted cautiously, as the included literature was largely retrospective and heterogeneous, and certainty assessments may not fully capture differences in confounding, variable definition, and outcome ascertainment across studies. It is important to note that the predominantly ‘low’ and ‘very low’ ratings in this review reflect the structural constraints of the GRADE framework, which inherently assigns lower certainty to retrospective and observational studies. While GRADE is a gold standard for clinical evidence, its application to the SDoH literature, which relies almost exclusively on observational data, may underrepresent the individual merit or internal validity for these population trends Thus, these ratings should be interpreted as a reflection of the challenge of achieving “high” certainty designations within traditional clinical hierarchies rather than as a lack of utility in the findings themselves.

Furthermore, race, socioeconomic status, and rurality are often intertwined, making it difficult to evaluate them as distinct variables. Retrospective reliance on ICD codes and the lack of patient-level SDoH further limits causal assessments. Many used area-level income proxies, and few accounted for patient-specific social determinants beyond race, which limits the ability to assess causality.

The central challenge in the literature is determining if treatment variations reflect sociodemographic bias or clinical necessity based. However, this review found evidence that tumor characteristics alone do not explain management trends. For example, as shown in the Race section of the results, Black patients were more likely to be managed with observation despite presenting with larger, more advanced tumors. This suggests that clinical severity does not always dictate treatment intensity uniformly across demographic groups. Despite this, because treatment plans are personal and each study is so heterogenous, findings cannot be interpreted with utter certainty.

Furthermore, the lack of explicit NF2 exclusion in 65% of the included studies is a notable limitation. Because NF2 patients typically present at a younger age and often require more complex management, their potential inclusion in these datasets could confound reported associations. Specifically, this ambiguity may skew findings related to age at diagnosis and treatment selection, as NF2 is a clinically distinct patient population. However, a very large majority of VS are sporadic, it made sense to include studies that did not address NF2 status. Future disparity research should utilize more granular coding to decouple these patient populations.

In summary, this review demonstrates that disparities in vestibular schwannoma care are multi-factorial, with race and insurance status showing the most consistent associations between management, outcomes, and disparities. As treatment continues to evolve and become more centralized, future work should prioritize multi-institutional studies to incorporate patient-level socioeconomic data and evaluate outcomes beyond management selection.

## Data Availability

No datasets were generated or analysed during the current study.

## References

[1] Goldbrunner R, Weller M, Regis J, Lund-Johansen M, Stavrinou P, Reuss D, Evans DG, Lefranc F, Sallabanda K, Falini A et al (2020) EANO guideline on the diagnosis and treatment of vestibular schwannoma. Neuro Oncol 22(1):31–45 31504802 10.1093/neuonc/noz153 PMC6954440

[2] Marinelli JP, Beeler CJ, Carlson ML, Caye-Thomasen P, Spear SA, Erbele ID (2022) Global Incidence of Sporadic Vestibular Schwannoma: A Systematic Review. Otolaryngol Head Neck Surg 167(2):209–214 34464224 10.1177/01945998211042006

[3] Reznitsky M, Petersen M, West N, Stangerup SE, Cayé-Thomasen P (2021) The natural history of vestibular schwannoma growth-prospective 40-year data from an unselected national cohort. Neuro Oncol 23(5):827–836 33068429 10.1093/neuonc/noaa230 PMC8099466

[4] Gupta VK, Thakker A, Gupta KK (2020) Vestibular Schwannoma: What We Know and Where We are Heading. Head Neck Pathol 14(4):1058–1066 32232723 10.1007/s12105-020-01155-x PMC7669921

[5] Marinelli JP, Grossardt BR, Lane JI, Carlson ML (2020) Rising incidence of sporadic vestibular schwannoma: true biological shift versus simply greater detection. Otol Neurotol 41(6):813–847 32150020 10.1097/MAO.0000000000002626 PMC7311288

[6] Pruijn IMJ, Kievit W, Hentschel MA, Mulder JJS, Kunst HPM (2021) What determines quality of life in patients with vestibular schwannoma? Clin Otolaryngol 46(2):412–420 33326685 10.1111/coa.13691 PMC7986908

[7] Cioffi G, Yeboa DN, Kelly M, Patil N, Manzoor N, Greppin K, Takaoka K, Waite K, Kruchko C, Barnholtz-Sloan JS (2004) Epidemiology of vestibular schwannoma in the United States, 2004–2016. Neuro-oncol Adv 2(1):vdaa135 10.1093/noajnl/vdaa135 PMC7672330 33241216

[8] Ostrom QT, Cioffi G, Gittleman H, Patil N, Waite K, Kruchko C, Barnholtz-Sloan JS (2019) CBTRUS Statistical Report: Primary Brain and Other Central Nervous System Tumors Diagnosed in the United States in 2012–2016. Neuro Oncol 21(Suppl 5):v1–v100 31675094 10.1093/neuonc/noz150 PMC6823730

[9] Babu R, Sharma R, Bagley JH, Hatef J, Friedman AH, Adamson C (2013) Vestibular schwannomas in the modern era: epidemiology, treatment trends, and disparities in management. J Neurosurg 119(1):121–130 23432451 10.3171/2013.1.JNS121370

[10] Stangerup SE, Caye-Thomasen P (2012) Epidemiology and natural history of vestibular schwannomas. Otolaryngol Clin North Am 45(2):257–268 vii 22483814 10.1016/j.otc.2011.12.008

[11] Carlson ML, Marston AP, Glasgow AE, Habermann EB, Sweeney AD, Link MJ, Wanna GB (2016) Racial differences in vestibular schwannoma. Laryngoscope 126(9):2128–2133 26917495 10.1002/lary.25892

[12] Quimby AE, Salmon MK, Zhao CH, Bigelow JYKL, Ruckenstein DC, Brant MJ (2024) Socioeconomic determinants impact quality of life at vestibular schwannoma diagnosis. J Clin Neurosci 119:122–128 38007900 10.1016/j.jocn.2023.11.028

[13] Matthew L, Carlson AEG, Brandon R, Grossardt, Elizabeth B, Habermann 3 MJ, Link (2016) Does where you live influence how your vestibular schwannoma is managed? Examining geographical differences in vestibular schwannoma treatment across the United States. J Neurooncol 199(2):269–279 10.1007/s11060-016-2170-5 27334903

[14] Schünemann HJ, Brennan S, Akl EA, Hultcrantz M, Alonso-Coello P, Xia J, Davoli M, Rojas MX, Meerpohl JJ, Flottorp S et al (2023) The development methods of official GRADE articles and requirements for claiming the use of GRADE - A statement by the GRADE guidance group. J Clin Epidemiol 159:79–84 37211327 10.1016/j.jclinepi.2023.05.010

[15] McClelland S 3rd, Kim E, Murphy JD, Jaboin JJ (2017) Impact of insurance status and race on receipt of treatment for acoustic neuroma: a national cancer database analysis. J Clin Neurosci 42:143–147 28343920 10.1016/j.jocn.2017.03.018

[16] McClelland S, Kim E, Murphy JD, Jaboin JJ (2016) Nonoperative management of acoustic neuroma in geriatric patients: a National Cancer Database analysis. J Radiat Oncol 5(4):345–349

[17] Carlson ML, Habermann EB, Wagie AE, Driscoll CL, Van Gompel JJ, Jacob JT, Link MJ (2015) The changing landscape of vestibular schwannoma management in the United States - a shift toward conservatism. Otolaryngol Head Neck Surg 153:440–446 26129740 10.1177/0194599815590105

[18] Desai AD, Shah VP, Tseng CC, Povolotskiy R, Wackym PA, Ying YM (2022) Impact of Social Determinants of Health on Stereotactic Radiotherapy for Vestibular Schwannoma. Laryngoscope 132(11):2232–2240 35076095 10.1002/lary.30016

[19] Hatch JL, Bauschard MJ, Nguyen SA, Lambert PR, Meyer TA, McRackan TR (2018) National Trends in Vestibular Schwannoma Surgery: Influence of Patient Characteristics on Outcomes. Otolaryngol Head Neck Surg 159(1):102–109 29584554 10.1177/0194599818765717 PMC6712975

[20] McClelland S 3rd, Guo H, Okuyemi KS (2011) Morbidity and mortality following acoustic neuroma excision in the United States: analysis of racial disparities during a decade in the radiosurgery era. Neuro Oncol 13(11):1252–1259 21856684 10.1093/neuonc/nor118 PMC3199160

[21] Ahmed OH, Mahboubi H, Lahham S, Pham C, Djalilian HR (2014) Trends in demographics, charges, and outcomes of patients undergoing excision of sporadic vestibular schwannoma. Otolaryngol - Head Neck Surg (United States) 150(2):266–274 10.1177/0194599813507234 24091426

[22] Bowers CA, Gurgel RK, Brimley C, Hawryluk GW, Taggart M, Braden S, Collett T, Gale D, Salzman KL, MacDonald JD (2016) Surgical treatment of vestibular schwannoma: does age matter? World Neurosurg 96:58–65 27565466 10.1016/j.wneu.2016.08.054

[23] Raymond M, Ghanouni A, Brooks K, Clark SM, Mattox DE (2021) Adherence to long-term follow-up in patients with sporadic vestibular schwannomas managed with serial observation. OTO Open 5(3):2473974X211036653 34396030 10.1177/2473974X211036653 PMC8358519

[24] Wang RS, Asfour L, Yang W, Zhang Y, Santacatterina M, Jethanamest D (2024) Patient Characteristics Impacting Adherence to Serial Observation for Vestibular Schwannomas. Otolaryngol Head Neck Surg 171(2):511–516 38520200 10.1002/ohn.729

[25] Fei-Zhang DJ, Sethia R, Abrahamson CW, Sosnoski OK, Sheyn AM, D’Souza JN, Chelius DC, Rastatter JC (2025) Individual- and Community-Level Social Determinant Associations With Acoustic Neuroma Disparities in the United States. Otol Neurotol 46(2):190–195 39627892 10.1097/MAO.0000000000004385

[26] Ellsperman SE, Bellile E, Fryatt R, Hoi K, Wang J, Fayson S, Banakis Hartl RM, Stucken EZ (2023) The Impact of Social Determinants of Health on Vestibular Schwannoma Management: A Single Institution Review. Otol Neurotol 44(5):507–512 37167450 10.1097/MAO.0000000000003883

[27] Brachimi E, Sooby P, Slim MAM, Kontorinis G (2024) The impact of multiple deprivation on the management of vestibular schwannomas. Eur Arch Otorhinolaryngol 281(8):4089–4094 38573514 10.1007/s00405-024-08570-8

[28] Bashjawish B, Kılıç S, Baredes S, Eloy JA, Liu JK, Ying YM (2019) Changing trends in management of vestibular schwannoma: a National Cancer Database study. Laryngoscope 129(5):1197–1205 30450631 10.1002/lary.27568

[29] Leon J, Trifiletti DM, Waddle MR, Vallow L, Ko S, May B, Tzou K, Ruiz-Garcia H, Lundy L, Chaichana K et al (2019) Trends in the initial management of vestibular schwannoma in the United States. J Clin Neurosci 68:174–178 31324471 10.1016/j.jocn.2019.07.002

[30] Ostler B, Killeen DE, Reisch J, Barnett S, Kutz JW Jr., Isaacson B, Hunter JB (2020) Patient demographics influencing vestibular schwannoma size and initial management plans. World Neurosurg 136:e440–e446 31931234 10.1016/j.wneu.2020.01.019

[31] Sonig A, Khan IS, Wadhwa R, Thakur JD, Nanda A (2005) The impact of comorbidities, regional trends, and hospital factors on discharge dispositions and hospital costs after acoustic neuroma microsurgery: a United States nationwide inpatient data sample study (2005–2009). Neurosurg Focus 33(3):E3 10.3171/2012.7.FOCUS12193 22937854

[32] Schwam ZG, Ferrandino R, Kaul VZ, Cosetti MK, Wanna GB (2019) National 30-day readmission and prolonged length of stay after vestibular schwannoma surgery: analysis of the Nationwide Readmissions Database. Am J Otolaryngol. 10.1016/j.amjoto.2019.102290 31530434

[33] Curry WT, Carter BS, Barker FG (2010) Racial, ethnic, and socioeconomic disparities in patient outcomes after craniotomy for tumor in adult patients in the United States, 1988–2004. Neurosurgery 66(3):427–437 20124933 10.1227/01.NEU.0000365265.10141.8E

[34] Cutri RM, Lin J, Wilson ML, Doherty JK, Pan DW (2010) Disparities in sporadic vestibular schwannoma initial presentation between a public safety net hospital and tertiary academic medical center at the same zip code 2010 to 2020. Ann Otol Rhinol Laryngol 133(6):605–612 10.1177/00034894241241201 38517145

[35] Patel S, Nuño M, Mukherjee D, Nosova K, Lad SP, Boakye M, Black KL, Patil CG (2013) Trends in surgical use and associated patient outcomes in the treatment of acoustic neuroma. World Neurosurg 80(1–2):142–147 22722042 10.1016/j.wneu.2012.06.029

[36] Mahboubi H, Ahmed OH, Yau AY, Ahmed YC, Djalilian HR (2014) Complications of surgery for sporadic vestibular schwannoma. Otolaryngol Head Neck Surg 150(2):275–281 24201062 10.1177/0194599813512106

[37] Patel AA, Kennedy D, Dupuis G, Levi JR, Weber PC (2024) Determining the Impact of Preoperative Psychiatric Comorbidities on Readmission After Resection of Vestibular Schwannoma. Otol Neurotol 45(8):e602–e606 39142317 10.1097/MAO.0000000000004277

[38] Homer AS, Kasthuri VS, Homer BJ, Jain R, Gall EK, Noonan KY (2024) The Association Between Medicaid Expansion and Disparities in Vestibular Schwannoma Incidence. Laryngoscope 134(10):4383–4388 38837793 10.1002/lary.31517

[39] Dicpinigaitis AJ, Kalakoti P, Schmidt M, Gurgel R, Cole C, Carlson A, Pickett B, Sun H, Mukherjee D, Al-Mufti F, Bowers CA (2021) Associations of Baseline Frailty Status and Age With Outcomes in Patients Undergoing Vestibular Schwannoma Resection. JAMA Otolaryngol Head Neck Surg 147:608–614 33914061 10.1001/jamaoto.2021.0670 PMC8085763

[40] Du EY, Assi SH, Moshtaghi O, Schwartz MS, Friedman RA, Dixon PR (2023) Socioeconomic Disparities in the Pursuit of Care at a High-Volume Institution for Surgical Resection of Vestibular Schwannomas. Otol Neurotol 44:826–832 37550886 10.1097/MAO.0000000000003975

[41] Ren Y, Sethi RKV, Stankovic KM (2020) National trends in surgical resection of vestibular schwannomas. Otolaryngol Head Neck Surg 163:1244–1249 32571146 10.1177/0194599820932148

[42] Aboueisha MA, Manayan R, Tie K, Issa PP, Al-Hamtary MA, Huang V, Naples JG (2024) Predictors of Prolonged Hospital Stay After Microsurgery for Vestibular Schwannoma: Analysis of a Decade of Data. Otol Neurotol 45:1159–1166 39284022 10.1097/MAO.0000000000004320

[43] Barker FG 2nd, Carter BS, Ojemann RG, Jyung RW, Poe DS, McKenna MJ (2003) Surgical excision of acoustic neuroma: patient outcome and provider caseload. Laryngoscope 113:1332–1343 12897555 10.1097/00005537-200308000-00013

[44] Butterfield JT, Golzarian S, Johnson R, Fellows E, Dhawan S, Chen CC, Marcotte EL, Venteicher AS (2022) Racial disparities in recommendations for surgical resection of primary brain tumours: a registry-based cohort analysis. Lancet 400:2063–2073 36502844 10.1016/S0140-6736(22)00839-X

[45] Kapoor S, Batra S, Carson K, Shuck J, Kharkar S, Gandhi R, Jackson J, Wemmer J, Terezakis S, Shokek O, Kleinberg L, Rigamonti D (2011) Long-term outcomes of vestibular schwannomas treated with fractionated stereotactic radiotherapy: an institutional experience. Int J Radiat Oncol Biol Phys 81:647–653 20884130 10.1016/j.ijrobp.2010.06.006 PMC4042401

[46] Kim KM, Lew RJ, Higashihara TJ, Yamashita S, Pang M, Stafford M, Goo C, Teehera KB, Luu K, Ho R, Carrazana E, Viereck J, Liow KK, Ghaffari-Rafi A (2024) Differences in tumor size, clinical, demographic, and socioeconomic profiles of central nervous system tumors among a racially diverse cohort: a retrospective case–control study. Surg Neurol Int. 10.25259/SNI_190_2024 39777174 PMC11704430

[47] Stepanidis K, Kessel M, Caye-Thomasen P, Stangerup SE (2014) Socio-demographic distribution of vestibular schwannomas in Denmark. Acta Otolaryngol 134:551–556 24655069 10.3109/00016489.2014.890293

[48] Sweeney AD, Glasgow AE, Link MJ, Habermann EB, Carlson ML (2016) Influence of Marital Status on Vestibular Schwannoma in the United States. Otol Neurotol 37:793–798 27203845 10.1097/MAO.0000000000001075

[49] Sylvester MJ, Shastri DN, Patel VM, Raikundalia MD, Eloy JA, Baredes S, Ying YM (2017) Outcomes of Vestibular Schwannoma Surgery among the Elderly. Otolaryngol Head Neck Surg 156:166–172 28045630 10.1177/0194599816677522

[50] Tang OY, Rivera Perla KM, Lim RK, Weil RJ, Toms SA (2021) The impact of hospital safety-net status on inpatient outcomes for brain tumor craniotomy: a 10-year nationwide analysis. Neurooncol Adv 3 10.1093/noajnl/vdaa167 PMC7813162 33506205

